# Dupilumab improved atopic dermatitis without aggravating tuberculous eosinophilic pleural effusion in hyperimmunoglobulin-E syndrome

**DOI:** 10.1016/j.jdcr.2024.04.003

**Published:** 2024-04-16

**Authors:** Piyaporn Chokevittaya, Irin Vichara-anont, Thanachit Krikeerati, Mongkhon Sompornrattanaphan, Ruchira Ruangchira-urai, Narissara Suratannon, Torpong Thongngarm, Chamard Wongsa

**Affiliations:** a Division of Allergy and Clinical Immunology, Department of Medicine, Faculty of Medicine Siriraj Hospital, Mahidol University, Bangkok, Thailand; b Department of Pathology, Faculty of Medicine Siriraj Hospital, Mahidol University, Bangkok, Thailand; c Faculty of Medicine, Division of Allergy, Immunology and Rheumatology, Department of Pediatrics, Center of Excellence for Allergy and Clinical Immunology, Chulalongkorn University, King Chulalongkorn Memorial Hospital, Thai Red Cross Society, Bangkok, Thailand

**Keywords:** atopic dermatitis, dupilumab, eosinophilic, HyperIgE, plueral effusion, tuberculosis

## Introduction

Hyperimmunoglobulin-E syndrome (HIES) typically manifests with severe atopic dermatitis (AD), recurrent pulmonary infections, and often eosinophilia. Eosinophilia observed during dupilumab treatment for severe AD in patients with HIES may be attributed to various factors, such as adverse effects of dupilumab, disease progression, or concurrent illnesses. The crucial decision in managing this scenario revolves around whether to continue or withhold dupilumab. In this context, we present a case where a patient with HIES developed a new-onset eosinophilic pleural effusion during dupilumab treatment for severe AD.

## Case report

**Fig 1:**
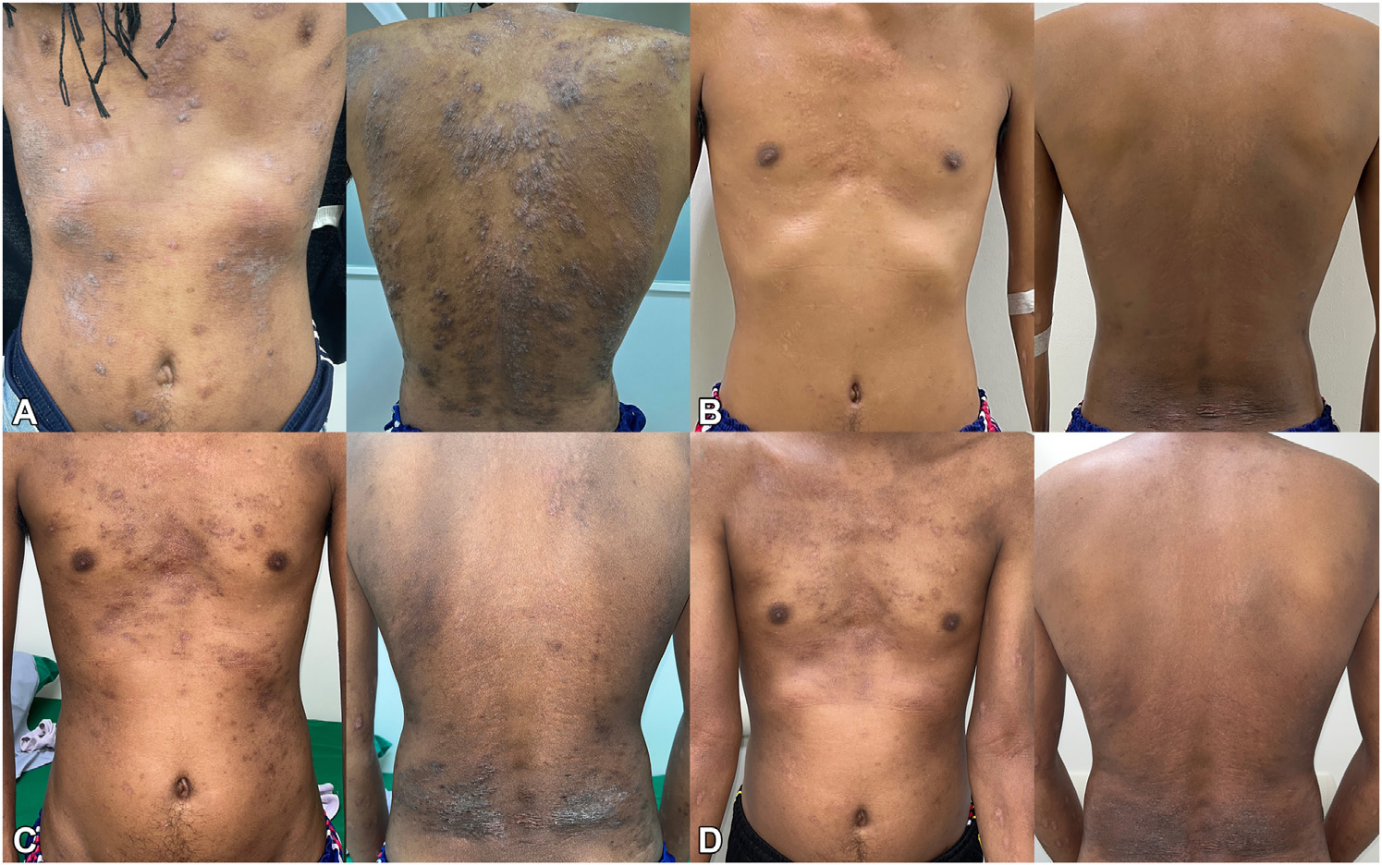
Progression of atopic dermatitis severity during dupilumab treatment. **A,** Baseline at the initiation of dupilumab. **B,** At the onset of tuberculosis (21 months after dupilumab initiation). **C,** Three months after withholding dupilumab. **D,** Three months after restarting dupilumab.

A 30-year-old man presented with severe AD, as shown in Fig 1, *A*, which had begun at the age of 20. He had a history of recurrent upper respiratory tract infections since childhood and exhibited distinctive features of HIES, including facial asymmetry, broad nose, deep-set eyes, prominent forehead, hyperextensibility of joints, and pectus excavatum. Laboratory tests showed markedly increased blood eosinophil count (BEC) of 3534 cells/μL (normal range: 0-600 cells/μL) and serum immunoglobulin E of 49,100 IU/mL (normal range: 0-100 IU/mL), with secondary causes excluded. The chest X-ray was normal.

Whole-genome sequencing revealed a novel heterozygous missense variant in the *STAT3* gene, c.1066C > G (p.[Pro356Ala]), which was identified as a variant of uncertain significance. The Mendelian Clinically Applicable Pathogenicity score of 0.110 suggests potential pathogenicity. Notably, another nucleotide in the same codon was established as pathogenic in autosomal dominant patients with HIES (c.1067C > G, p.Pro356Arg).^1^ Hence, HIES with *STAT3* mutation was diagnosed in this patient.

**Fig 2:**
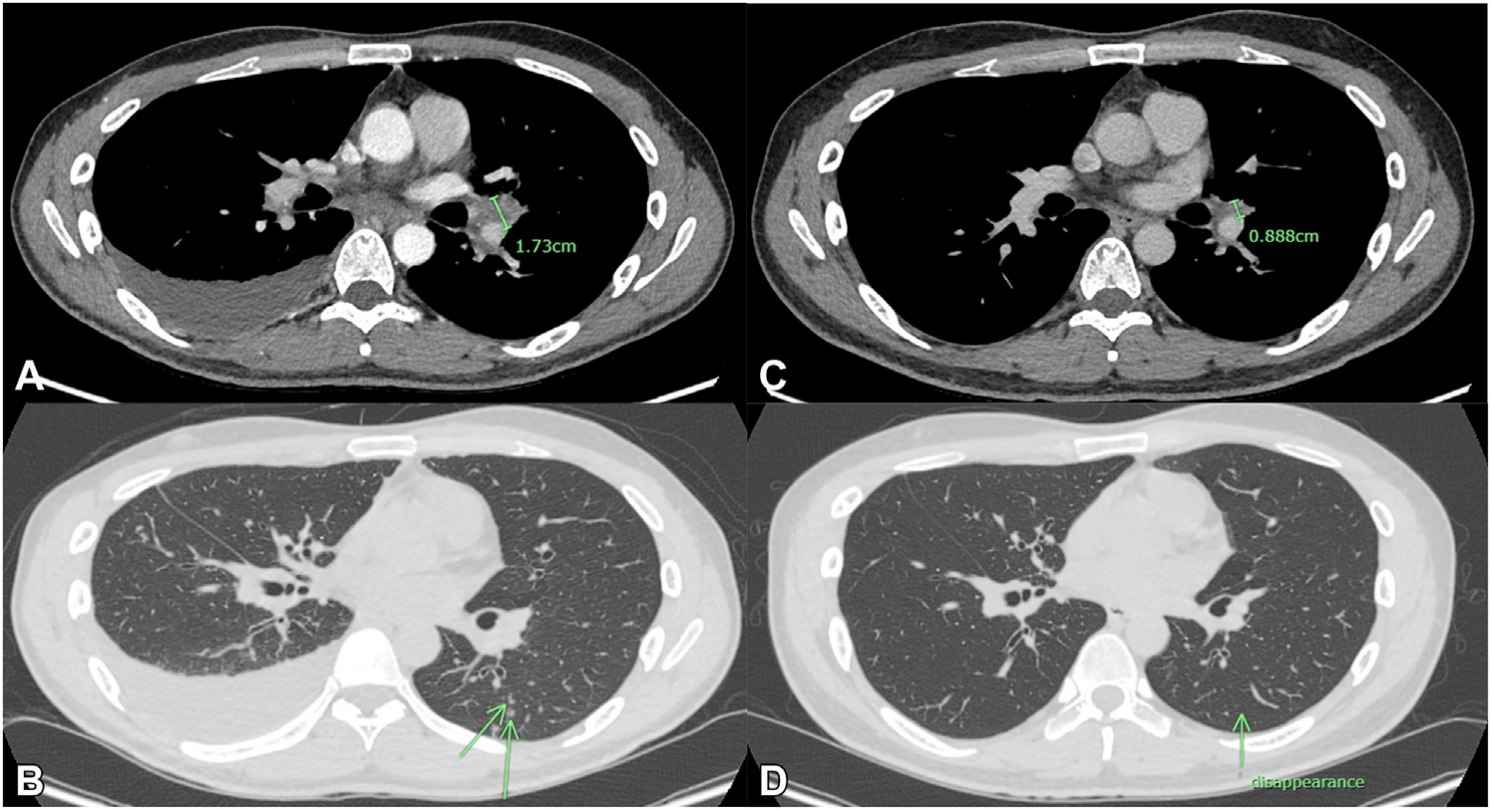
Chest computed tomography demonstrated. **A,** Right pleural effusion with hilar lymphadenopathy at baseline. **B,***Arrow* shows multiple areas of centrilobular nodules in a tree-in-bud pattern. **C,** Pleural effusion was resolved, and the size of the hilar lymph node significantly decreased after a complete course of antituberculosis treatment. **D,** Pulmonary nodules disappeared.

A skin biopsy showed spongiotic dermatitis without eosinophil or abnormal cell infiltration. Despite various conventional treatments, severe AD remained uncontrolled. Dupilumab led to significant improvement after 4 doses, and its effectiveness remained persistent after 21 months of treatment (Fig 1, *B*). However, he subsequently developed a low-grade fever, nonproductive cough, pleuritic chest pain, and a new onset of right pleural effusion. Pleural fluid analysis revealed an exudative profile with 44% eosinophils and adenosine deaminase levels of 80 U/L, while BEC of 1410 cells/μL. An abnormal chest computerized tomography scan is demonstrated in Fig 2, *A* and *B*.

**Fig 3:**
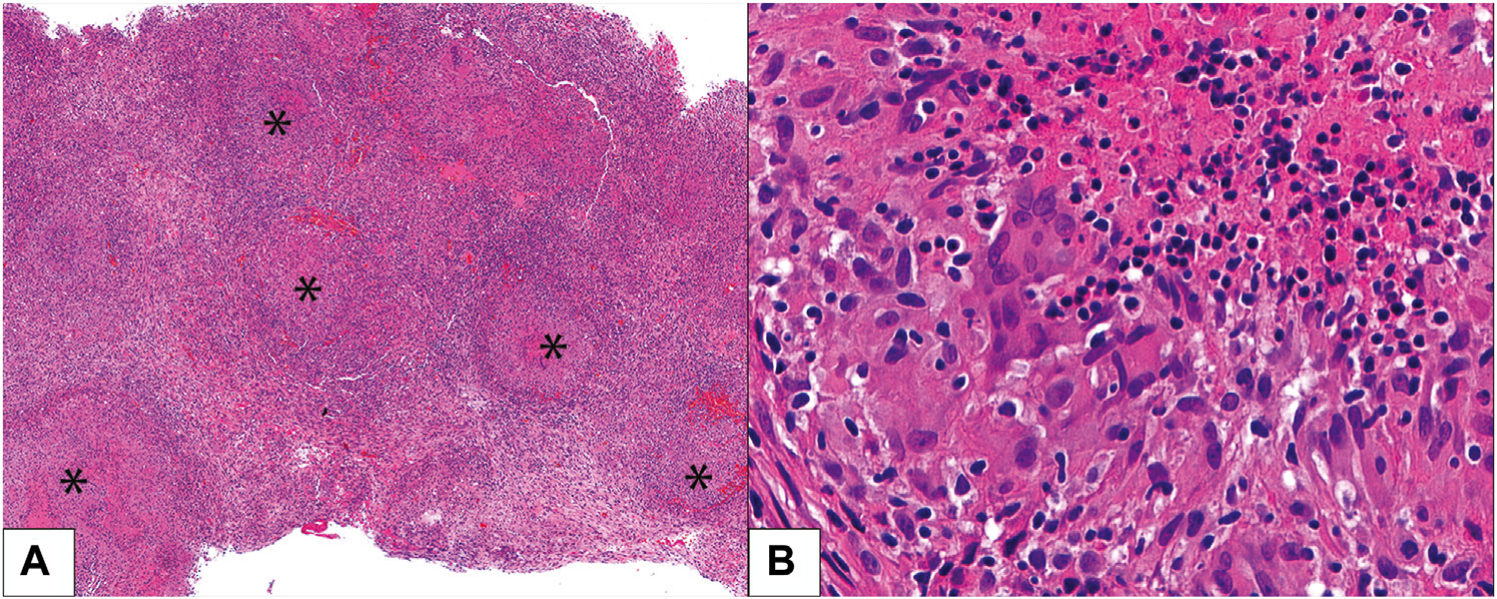
Pathology of pleural tissue showing diffuse necrotizing granulomatous inflammation and eosinophils were minimally present. **A,** Scattered aggregates of epithelioid histiocytes with central necrosis (*asterisk*; H&E, 200×). **B,** Edge of the necrosis rimmed by epithelioid histiocytes having oval nuclei with abundant eosinophilic cytoplasm and indistinct cell borders (H&E, 600×).

Pleuroscopy with biopsy revealed diffuse necrotizing granulomatous inflammation in the pleural tissue, consistent with *Mycobacterium tuberculosis* infection (Fig 3). No causative organisms or malignancies were identified from the tissue section or cultures of pleural effusion. Antineutrophil cytoplasmic antibodies were negative. Based on the consistent pathological findings and the high diagnostic specificity for tuberculosis (TB) of the adenosine deaminase level exceeding 40, the diagnosis of pulmonary TB and TB pleuritis was reasonably established.^2^ The absence of TB growth in culture, which could be observed in 44% of TB pleuritis cases, did not refute the diagnosis.^2^ Consequently, anti-TB therapy was started, and dupilumab was discontinued due to concerns regarding its potential to induce eosinophilic pleural effusion.

**Fig 4:**
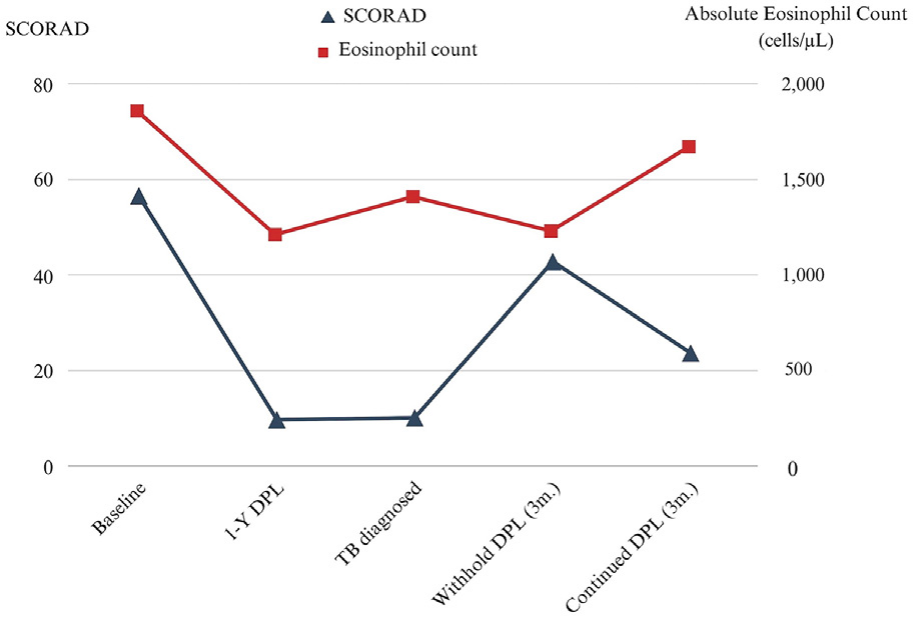
The SCORing Atopic Dermatitis index and absolute eosinophil count during dupilumab treatment. *DPL*, Dupilumab; *m*, months; *SCORAD*, The SCORing Atopic Dermatitis index; *TB*, tuberculosis.

The patient exhibited a favorable response to anti-TB treatment (Fig 2, *C* and *D*), which was sustained despite the subsequent resumption of dupilumab therapy 3 months later following an AD flare (Fig 1, *C*). Notably, within 3 months of restarting dupilumab, the patient's skin lesions markedly improved (Fig 1, *D*) with no recurrence of pleural effusion. Based on all available evidence, it is suggested that the improvement in pleural effusion is primarily attributed to TB treatment rather than the temporary discontinuation of dupilumab. The correlation between AD severity and BEC throughout the treatment course is illustrated in Fig 4.

## Discussion

Eosinophilia can be induced by dupilumab itself, typically transient and rarely resulting in clinical symptoms. The potential mechanism for dupilumab-induced eosinophilia involves the inhibition of eosinophil migration to tissues by blocking vascular cell adhesion molecules-1.^3^ Across all dupilumab studies, the incidence of eosinophilia ranged from 0% to 13.6%, with the lowest occurrences observed in AD studies.^3^ According to the phase 3 dupilumab trials in patients with AD, asymptomatic increased BEC at weeks 4 to 12, followed by a decline to baseline or lower at weeks 12 to 16, could be expected with no serious adverse events.^3^ No cases of eosinophilic pleural effusion during dupilumab treatment for severe AD have been reported to date. However, a German patient with severe asthma experienced a significant rise in BEC and eosinophilic pleural effusion 6 weeks after initiating dupilumab treatment.^4^ Other potential causes of eosinophilic pleural effusion include malignancy, TB, pulmonary embolism, or idiopathic causes.^5^ Comprehensive investigations tailored to the patient’s conditions are necessary for accurate diagnosis.

While caution was advised regarding the use of dupilumab in cases of severe asthma with BEC surpassing 1500 cells/μL, a similar recommendation was not applied to severe AD.^6^ Considering the BEC, severity of AD, and the onset of chest symptoms in our patient, it is more likely that the recently observed eosinophilic pleural effusion is attributable to alternate etiologies other than dupilumab adverse effects. We, therefore, suggest monitoring complete blood count during dupilumab treatment, especially in patients with elevated BEC.

Treatment with dupilumab up to 4 years for adults with moderate-to-severe AD was not associated with an increased risk of systemic or cutaneous infection.^7^ Four reported cases of HIES with *STAT3* mutation were successfully treated with dupilumab for their severe AD without any serious adverse events.^8^^,^^9^ Although TB was not a common infection in patients with HIES and *STAT3* mutations, evidence indicates potential underlying mechanisms contributing to such infection. Wang et al reported that *STAT3* polymorphism could potentially enhance susceptibility to TB through diverse mechanisms.^10^ According to their findings, STAT3 down-regulation reduces the ability of innate immunity to control mycobacterial infection, particularly in macrophages, leading to a decrease in downstream signaling of multiple antimycobacterial pathways, such as autophagy and phagosome/nitric oxide killing.^10^ However, the precise relationship between TB and *STAT3* mutation still needs further studies. To date, there has been no recommendation to screen for latent TB before starting dupilumab. However, it should be considered in susceptible patients, such as those with immunodeficiency or those residing in TB-endemic areas.

In conclusion, patients who develop eosinophilic organ involvement during dupilumab treatment should undergo extensive investigations. The host immunity and the temporal relationship between the onset of symptoms and the timing of dupilumab administration should be considered in diagnosis. Based on our patient, dupilumab was safe and successfully reduced AD severity during anti-TB treatment.

## Conflicts of interest

Dr Sompornrattanaphan has received honoraria for scientific lectures from A. Menarini, Astra-Zeneca, GSK, Takeda, and Viatris. Dr Thongngarm has received honoraria for scientific lectures from A. Menarini, Astra-Zeneca, GSK, Novartis, Sanofi, Takeda, and Viatris and served on the advisory boards for Sanofi and Viatris. Dr Wongsa, has received honoraria for scientific lectures from A. Menarini, Astra-Zeneca, GSK, Takeda, and Viatris. Drs Chokevittaya, Vichara-anont, Krikeerati, Ruangchira-urai, and Suratannon have no conflicts of interest to declare.
